# Serum Analysis of Women with Early-Stage Breast Cancer Using a Mini-Array of Tumor-Associated Antigens

**DOI:** 10.3390/bios10100149

**Published:** 2020-10-21

**Authors:** Alma Rosa Oaxaca-Camacho, Oscar René Ochoa-Mojica, Adriana Aguilar-Lemarroy, Luis F. Jave-Suárez, José Francisco Muñoz-Valle, Eduardo Padilla-Camberos, Juan Antonio Núñez-Hernández, Sara E. Herrera-Rodríguez, Moisés Martínez-Velázquez, Ahtziri Socorro Carranza-Aranda, José Alfonso Cruz-Ramos, Abel Gutiérrez-Ortega, Rodolfo Hernández-Gutiérrez

**Affiliations:** 1 Centro de Investigación y Asistencia en Tecnología y Diseño del Estado de Jalisco, A.C. (CIATEJ), 44270 Guadalajara, Mexico; almave15@hotmail.com (A.R.O.-C.); oscarene8am@gmail.com (O.R.O.-M.); epadilla@ciatej.mx (E.P.-C.); nanojanh8@gmail.com (J.A.N.-H.); sherrera@ciatej.mx (S.E.H.-R.); mmartinez@ciatej.mx (M.M.-V.); aortega@ciatej.mx (A.G.-O.); 2 Centro de Investigación Biomédica de Occidente (CIBO), División de Inmunología, Instituto Mexicano del Seguro Social (IMSS), 44340 Guadalajara, Mexico; adry.aguilar.lemarroy@gmail.com (A.A.-L.); lfjave@gmail.com (L.F.J.-S.); 3 Centro Universitario de Ciencias de la Salud, Universidad de Guadalajara, 44340 Guadalajara, Mexico; drjosefranciscomv@cucs.udg.mx (J.F.M.-V.); ahtziricarranza19@gmail.com (A.S.C.-A.); josealfonsocr@gmail.com (J.A.C.-R.); 4 Instituto Jalisciense de Cancerología (IJC), Departamento de Enseñanza, Capacitación e Investigación, 44280 Guadalajara, Mexico

**Keywords:** printed mini-array, tumor-associated antigens, early-stage breast cancer

## Abstract

*Background:* Several studies have shown that patients with cancer have antibodies in serum that react with cellular autoantigens, known as Tumor-Associated Antigens (TAA). The present work aimed to determine whether a mini-array comprising four recombinant TAA increases the detection of specific serum antibodies for the diagnosis of early-stage breast cancer. *Methods:* The mini-array included Alpha 1-AntiTrypsin (A1AT), TriosePhosphate Isomerase 1 (TPI1), Peptidyl-Prolyl cis-trans Isomerase A (PPIA), and PeroxiReDoXin 2 (PRDX2) full-length recombinant proteins. The proteins were produced after gene cloning, expression, and purification, and were verified by Western blot assays. Then, Dot-Blot was performed to find antibodies against the four TAA in 12 sera from women with early-stage breast cancer (stage II) and 12 sera from healthy women. *Results:* Antibody detection against individual TAA in early-stage breast cancer sera ranged from 58.3% to 83.3%. However, evaluation of the four TAA showed that there was a positive antibody reaction reaching a sensitivity of 100% and a specificity of 85% in early-stage breast cancer, suggesting that this mini-array must be evaluated as a clinical diagnostic tool for early-stage breast cancer in a larger sample size. *Conclusion:* Our results suggest that TAA mini-arrays may provide a promising and powerful method for improving the detection of breast cancer in Mexican women.

## 1. Introduction

Breast cancer ranks first worldwide in frequency among all types of cancer found in women. The number of cases and deaths from breast cancer has increased importantly, from 1.15 and 0.410 million in 2002 to 2.088 and 0.626 million in 2018, respectively [1,2,3,4,5,6]. The prognosis of breast cancer is correlated with the stage at which the disease is diagnosed, and the gold standard (test) for diagnosis is mammography screening, which has been employed during the last 20 years as a key tool in the control of breast cancer, with 65–80% sensitivity and specificity [7,8]. During the last 15 years, the mortality rates of breast cancer have been slightly contained due to earlier detection and more timely and effective therapies; nevertheless, early detection could be faster and more effective with the use of biomarkers in rapid detection methods.

The detection of breast cancer at its earliest stages could contribute to a considerable and effective reduction worldwide in morbidity and mortality rates caused by this type of cancer [9]. Therefore, it is of the utmost importance, and there is a great need to develop, test, validate, and place new detection and diagnostic tests that are minimally invasive or noninvasive, simple, and inexpensive on the market for use in the field and tests that do not require expensive and sophisticated equipment. In Mexico, only around 14–25% of women have access to a mammography analysis; thus, it is very important to develop a rapid, specific, sensitive, and simple method for a better and accessible test that can be utilized as a screening method that aids in or is complementary to mammography.

If breast cancer is detected and therapy is applied when the tumor is confined to the breast, remission can reach nearly 100%. Unfortunately, small breast tumors are hardly detected by physical examination in early stages and, sometimes, they may not be observed in a mammography analysis, particularly in young women and women with dense breast tissue [10,11,12]. 

At present, the search and discovery of biomarkers and the development of new methods to detect breast cancer in asymptomatic conditions would help to reduce morbidity and mortality in a considerable manner. Molecular markers can be a very useful and reliable tool for the detection of early cancer, speeding up treatment to reduce morbidity, mortality, psychological effects, and economic costs. For several years, researchers have had a special interest in discovering new biomarkers for the detection of early breast cancer. Currently, some reports demonstrate that some specific molecules, such as p53, Heat-Shock Protein (HSP) 90, c-erbB-2/HER2/neu (HER2), mucin-related protein, and RS/DJ-1, trigger a self-immune response, which results in the production of autoantibodies. Serological autoantibodies are detected prior to the presence of cancer symptoms and signs. Therefore, autoantibody presence and concentration in a patient’s serum have been regarded as a diagnostic biomarker for the diagnosis of early-stage cancer [13]. However, the presence of antibodies against these proteins has been observed only in 15–45% of patients with breast cancer, leading to the search for novel and better markers for the diagnosis of this pathology [10,11,12,13].

The humoral immune response produces antigen-specific antibodies against some self-proteins denominated Tumor-Associated Antigens (TAA), which may be formed through different mechanisms, such as post-translational modifications or the overexpression of immunologically relevant proteins [11,12,13,14].

Breast cancer is a disease with special peculiarities, in that it is highly heterogeneous and because cancer cells can express a great variety of TAA that are capable of stimulating an immune response (antibody production) in patients. Interestingly, this immune response can appear several months or even years before the clinical signs and diagnosis of this malignancy [15,16].

TAA and their antibodies may reflect an in-vivo intensification of an early carcinogenic signal; therefore, they might permit earlier detection of cancer, alone or combined with currently used techniques. Human fluids, such as saliva, blood, urine, prostatic and vaginal secretions, and bone marrow, are considered ideal sources in which to assess the presence of cancer biomarkers, because they are easy to obtain and the techniques are non- or minimally invasive [17,18]. Serum, in particular, contains several circulating antigens and antibodies related to cancer status, progression, and development [19,20,21,22,23,24,25,26,27].

Antibodies comprise one of the typical molecules of autoimmune diseases, such as systemic scleroderma, rheumatoid arthritis, and systemic lupus erythematosus, in addition to cytokines. Research for several decades has demonstrated that autoantibodies are very important biomarkers in clinical diagnosis and prognosis and are also useful for targeting autoantigens and for the development of novel methods. The humoral response, characteristic of some autoimmune diseases, is similar to the immune mechanisms triggered by cancer and it is related to chronic inflammation [28,29,30].

Within the area of cancer research, Tumor Antigen-Antibodies (TA-Ab) have been described in early- and late-stage disease for many types of human tumors. These autoantibodies represent an immunological response to proteins of the cytoplasmic membrane, the nuclear membrane, and other proteins or components of cancer cells. Autoantibodies are indicative of the recognition of the immune system of tumor cells, secondary to an abnormal presentation of antigens by cancer cells to immune system cells; some authors assert that the generation of autoantibodies in a patient with cancer is associated with reactivity and immune surveillance. Autoantibodies are very stable proteins and their presence in serum is long-lasting, even when the antigen has disappeared [28,29,30]. Histological examination of cancer tissue samples reveals the presence of T and B cells near and around the tumor. These cells participate in immune surveillance and signal amplification to produce antibodies [30].

In the present study, as a preliminary analysis, four TAA were used as printed antigens by Dot-Blot for detecting specific autoantibodies against these four recombinant antigens. Briefly, 12 sera obtained from subjects with early-stage (stage II) breast cancer and 12 sera from healthy volunteers were evaluated for autoantibody detection.

## 2. Materials and Methods

### 2.1. Serum Samples

In the present study, sera from 12 patients with breast cancer in early stages and sera from 12 healthy humans were provided by the Division of Oncology and Hematology of the Instituto Mexicano del Seguro Social (IMSS). Cancer sera were collected at same time of diagnosis and when patients had not yet received any type of treatment, e.g., chemotherapy or radiotherapy. Normal human sera were collected from subjects who had no evidence of malignancy by breast mammography during health examinations. None of the patients nor healthy control subjects had other malignancies or any autoimmune disease. The study was approved by the IMSS Institutional Review Board and all patients signed informed consent to participate in the study. In this preliminary study, sera from 12 patients with cancer and sera from 12 healthy persons were employed, with both groups having a comparable mean age of 44.17 and 45.83 years, respectively. Briefly and in general, breast cancer is classified in four stages. Stage 1 breast cancer means that the cancer is small and only in the breast tissue, or it might be found in lymph nodes close to the breast. Stage 2 breast cancer means that the cancer is either in the breast or in the nearby lymph nodes or both (Stages 1 and 2 are defined as early-stage breast cancer). Stage 3 means that the cancer has spread from the breast to lymph nodes close to the breast or to the skin of the breast or to the chest wall, while Stage 4 breast cancer means that the cancer has spread to other parts of the body.

### 2.2. Expression and Purification of Recombinant Tumor-Associated Antigens (TAA)

Four Tumor-Associated Antigens (TAA) were selected for the present study, comprising TPI-1 (UniProtKB ID: P60174), PRDX-2 (UniProtKB ID: P32119), PPIA (UniProtKB ID: P62937), and A1AT (UniProtKB ID: P01009) proteins. The A1AT protein was previously reported by our group [31]. On the other hand, TPI-1, PRDX-2, and PPIA were reported by other authors as TAA [32]. These four proteins were selected for cloning and expression in recombinant form in *Escherichia coli*. In brief, TPI-1, PRDX-2, PPIA, and A1AT Open Reading Frames (ORF) were cloned into the vector pCR2.1-TOPO; after this, they were subcloned into pTrcHis expression plasmid (Thermo Fisher Scientific). Afterward, the constructed plasmids were mobilized into the *E. coli* BL21 strain and recombinant proteins were synthesized and purified through immobilized metal-affinity chromatography. The following purification process was carried out. Briefly, cell lysis was performed by means of five cycles of ultrasonication; the clarification of *E*. *coli* BL21 lysate in the following binding buffer: 20 mM sodium phosphate and 500 mM NaCl. Three washing steps were performed as follows: with 50 mM; with 100 mM, and with 200 mm of Imidazole. The elution step was conducted with 500 mM of Imidazole. The purified proteins were electrophoresed by sodium dodecyl sulfate polyacrylamide gel electrophoresis (SDS-PAGE) in preparative gels, and the proteins bands were obtained from gels and electroeluted (by using Electro-Eluter 422, BioRad, Hercules, Clearwater, FL, USA). Electroeluted proteins were quantified and utilized for blotting in the mini-array. The expression of recombinant purified proteins was examined by SDS-PAGE assay and Coomassie Blue staining.

### 2.3. Western-Blot Assays

Western-blot assays were carried out to confirm that the recombinant Tumor-Associated Antigens (TAA) protein bands observed in SDS-PAGE were recognized by an anti-Histidine antibody (Bio-Rad) and by specific antibodies for each protein. Expressed and purified recombinant proteins (TPI-1, PRDX-2, PPIA, and A1AT) were diluted individually in a Phosphate buffered saline PBS pH 7.0 solution at a final concentration of 0.5 µg/mL, as determined by a NanoDrop 2000 UV-VIS Spectrophotometer, electrophoresed in 10% or 12% SDS-PAGE, and were then electrotransferred to NitroCellulose Membranes (NCM). The NCM were blocked in PBS solution containing 0.05% Tween 20 (PBS-T) and 5% milk for 1 h at 28 °C with gentle rocking, then incubated for 18 h at 4 °C with anti-Histidine antibody (Bio-Rad) diluted 1:3000 in PBS-T, and finally incubated with HRP-conjugated goat anti-mouse IgG as secondary antibody diluted at 1:3000 for 4 h, followed by washing with PBS-T solution. Immunoreactive bands were detected using the HRP Conjugate Substrate kit (Bio-Rad). Western blots with specific antibodies were performed. The antibodies included mouse anti-A1AT (Sigma-Aldrich, USA), anti-TPI-1; anti-PRDX-2, and anti-PPIA supplied by Abnova Corporation (USA). These specific antibodies were raised against the full-length protein and employed at a 1:2000 dilution, while anti-mouse IgG-HRP secondary antibody was used at a 1:3000 dilution. Immunoreactive bands were detected using the HRP Conjugate Substrate kit (Bio-Rad).

### 2.4. Dot-Blot Assays

Dot-Blot assays were performed by using a Bio-Dot SF Microfiltration Apparatus (Bio-Rad) to evaluate the presence of antibodies in the patient’s sera as a blotting assay. Purified and quantified recombinant proteins were tested at a concentration of 200 ng per line; this was carried out as described as follows: 200 ng of each protein were suspended in 400 microliters of PBS pH 7.0, were placed in each well of the Bio-Dot SF Apparatus, and were subjected to vacuum pressure for 20 min, which allowed the total protein to be placed onto the nitrocellulose membrane. Next, the NCM were cut into strips, blocked with PBS-T20-Milk, and afterward were incubated with the individual patient’s serum for simultaneous detection of autoantibodies against the four recombinant TAA.

Briefly, NCM strips with blotted proteins and the control were incubated individually in Mini Incubation Trays (Bio-Rad) with each serum (individually) as follows: at room temperature with gentle rocking during 4 h; after that, with gentle rocking at 4 °C overnight (12 h) with the patient’s serum diluted 1:25 in PBS-T20-milk, and, at the end of the incubation, the strips of NCM were washed five times with PBS-T20 and were later incubated with HRP-conjugated goat anti-human IgG as secondary antibody diluted 1:2000 in PBS-T20-milk for 4 h at room temperature, followed by washing with PBS-T20 solution. Finally, immunoreactive bands were evidenced using the HRP Substrate and Detection kit (Opti-4CN, Bio-Rad).

## 3. Results

As described previously, women underwent screening mammograms to find breast cancer early. These results are summarized in Table 1 and Table 2.

### 3.1. Breast Cancer: TNM Staging

Current classification for solid tumors is based on the characteristics of their extension. Size of the primary tumor and presence of metastatic regional lymph nodes and/or of distant metastases are the key elements for tumor categorization. The Tumor-Node-Metastasis (TNM) system describes the amount and spread of cancer in a patient’s body: T describes the size of the tumor and any spread of the cancer into nearby tissue; N describes the spread of cancer to nearby lymph nodes, and M describes metastasis. The TNM staging system is the most common way that physicians stage breast cancer. The size is divided again as follows: T1, less than 2 cm; T2, from 2 to 5 cm, and T3, more than 5 cm. The spread of cancer lymph nodes is divided into N0–N3. The spread of cancer or not to other parts of the body is divided as follows: M0 cancer has not spread, and M1 cancer has spread to other parts of the body.

In the present study, a mini-array of four recombinant TAA was performed by blotting antigens over NCM and then probing with sera from 12 patients with stage-II breast cancer and sera from 12 healthy persons, in order to assess the presence of specific antibodies against the recombinant TAA. In this manner, individual and cumulative interaction between the sera and the TAA is shown.

### 3.2. Manufacture of the Mini-Array

**Figure 1:**
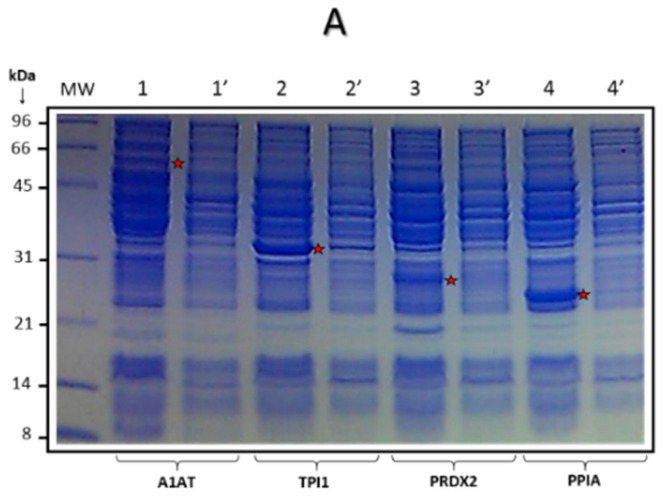

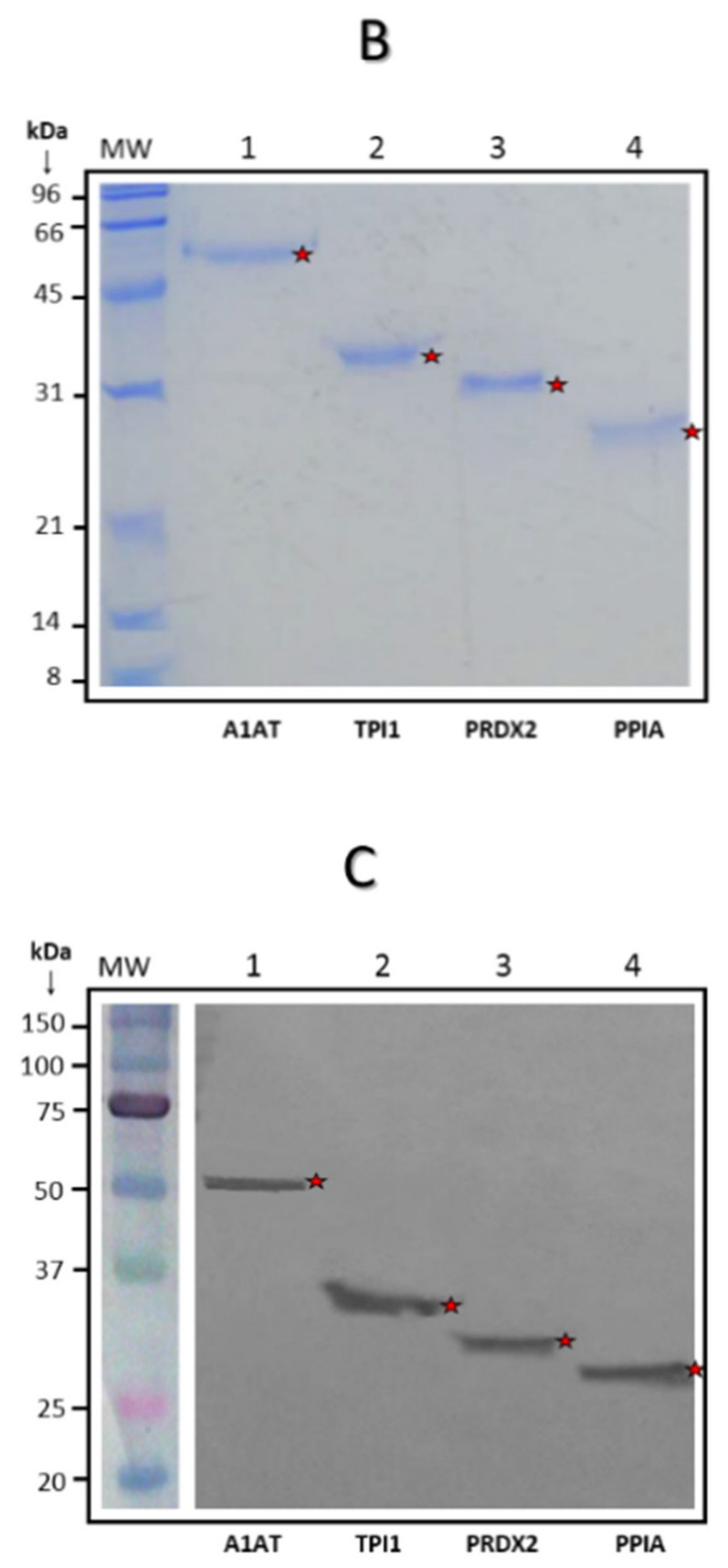
Expression and purification of recombinant Tumor-Associated Antigens TAA. (**A**) Synthesis of four recombinant proteins (TAA) in *Escherichia coli*. A1AT, TPI1, PRDX2, and PPIA; IPTG positive lanes **1**, **2**, **3**, and **4,** respectively, and **1′**, **2′**, **3′**, and **4′** IPTG negative lanes. (**B**) Histidine-tagged recombinant proteins were purified using affinity nickel column chromatography; electrophoresed proteins were detected by SDS-PAGE with Coomassie Blue staining. Lane 1, A1AT; Lane 2, TPI1; Lane 3, PRDX2, and Lane 4, PPIA. (**C**) Western-blot assays show the immunoreactivity of four recombinant proteins with anti-histidine antibody. Lane 1, A1AT; Lane 2, TPI1; Lane 3, PRDX2, and Lane 4, PPIA.

The Tumor-Associated Antigens (TAA) mini-array elaborated and used in this study included A1AT, TPI-1, PRDX-2, and PPIA full-length purified recombinant proteins. The four Histidine-Tagged (6His-Tag) recombinant proteins were expressed successfully in *E*. *coli* (Figure 1A). These proteins were purified by immobilized metal affinity chromatography (Figure 1B). In addition, the presence of recombinant proteins was determined by Western blot employing either an anti-histidine polyclonal antibody (Figure 1C) or specific antibodies against each protein (data not shown). The purified proteins were quantified by Bradford assay. After this, three different amounts of the proteins were evaluated with anti-his antibody (750, 500, and 200 ng), selecting the amount of 200 ng of protein-per-line for blotting over NCM (mini-array).

### 3.3. Presence of Antibodies Interacting with TAA Blotted on the Mini-Array

The sera of the patients were added separately to mini-tray incubation channels containing the strip blotted with the four recombinant purified proteins and an additional positive control line blotted with human IgG. A medium-to-strong recognition signal on the NCM was considered as positive, while a faint signal was considered as negative.

**Figure 2:**
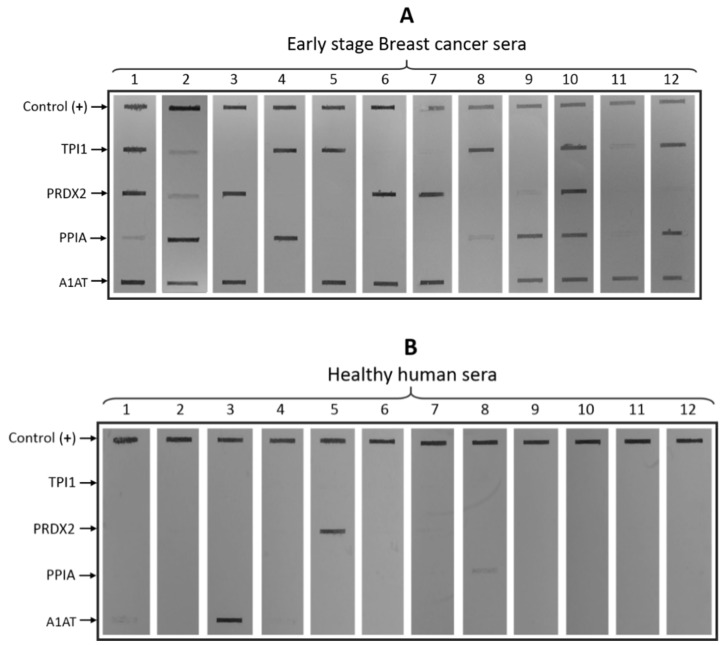
Dot-blot analysis for serum reactivity. (**A**) Early-stage breast cancer sera (lanes 1–12); top line, control lane (human IgG). The TPI1, PRDX2, PPIA, and A1AT, lanes 1–12, the patients’ sera. (**B**) Healthy human sera; top line, control lane (human IgG). TPI1, PRDX2, PPIA, and A1AT; lanes 1–12, healthy persons’ sera.

The frequency of antibody detection to an individual TAA in breast-cancer samples ranged from 50% to 83%. Sera from the 12 patients with breast cancer had antibodies against at least two TAA. Eight sera were positive for two TAA (lines 3–9 and line 11; Figure 2A), one serum was positive for three TAA (line 12; Figure 2A), and three sera were positive for four TAA (lines 1, 2, and 10, Figure 2A). The highest frequency of antibodies to an individual TAA in breast cancer was against A1AT (83.3%) and TPI1 (66.7%), followed by PPIA (58.3%) and PRDX2 (50%). Considering all four antigens, the value reached 100%; in fact, TPI1 and A1AT combined reached 100%. In contrast, the detection and reactivity of healthy human sera to the proteins in the mini-array was very low. Only two sera were positive (lines 3 and 5, for A1AT and PRDX2, respectively; Figure 2B) and two sera had a weak signal (lines 1 and 8, for A1AT and PPIA, respectively; Figure 2B).

One hundred percent of the 12 sera analyzed from patients with breast cancer were shown to have antibodies against at least two of the four TAA, which was significantly higher than the frequency found in sera from healthy subjects. In this preliminary study, the sera from 12 patients with cancer and from 12 healthy persons were used, with both groups having a similar mean age of 44.17 and 45.83 years, respectively. The status of breast cancer in TNM classification and pathology are summarized in Table 1. For the healthy individuals, the characteristics are summarized in Table 2.

### 3.4. Evaluation of Diagnostic Values of the Mini-Array with Four TAA for the Diagnosis of Breast Cancer

The relevance of a diagnostic test is based on its capacity to distinguish between a situation of disease and another of health. Then, the following question arises: Is this distinction possible by means of an approach of antibody detection with a single mini-array or a multiple mini-array comprising several recombinant TAA? To answer this, the data obtained from immunoreactivity against TPI1, PRDX2, PPIA, A1AT, and all together (according to the mini-array results and described in Table 1 and Table 2) were analyzed utilizing a chi-square (Fisher exact test). Specificity, sensitivity, positive predictive value, and negative predictive value were obtained, and the results were the following: for individual proteins, the results ranged from 0.5833 to 0.8333, while negative Predictive Values (*-*PV) ranged from 0.8333 to 1.0. For all four antigens, the +PV was 0.6667 and the -PV was 0.9167. Furthermore, the *p* value ratio for individual proteins was 0.072–0.0013 and 0.0001 for the four proteins (Table 3).

Taken together, these data demonstrate the usefulness of the multiple recombinant TAA mini-array, which could be relevant for increasing the clinical diagnostic quality of breast cancer. The data obtained with this study support previous indications and hypotheses on the detection of serum autoantibodies for the diagnosis of some types of cancer that could be enhanced by using a mini-array composed of several specific recombinant TAA as target antigens.

## 4. Discussion

Several studies have shown that a single biomarker could not completely distinguish between patients with cancer and healthy subjects. However, the combination of several biomarkers in a single test may offer a potential tool for the early detection of breast cancer and other types of cancers. On the other hand, the multistep nature of the tumorigenesis and the molecular pathogenesis of human malignancies renders detection at an early stage difficult. Therefore, it is very important to seek new options to achieve an early detection. Some previous studies indicated that combinations of multiple antigen/antibody systems might yield a higher sensitivity for the diagnosis of cancer [12,26,27], including the work by Wang et al. [33] on autoantibody signatures for cancer. The aim of our group was to increase both the specificity and sensitivity of autoantibodies as biomarkers in early-stage breast cancer by employing TAA arrays that include new or uncommon antigens. In this manner, it is possible to be more selective with a specific cancer, such as breast cancer, and not with others. The ultimate goal is to use the mini-array of the TAA reported in this work as a novel, noninvasive approach to identify cancer in normal or asymptomatic subjects, as well as in high-risk groups, specifically, for Mexican populations. We used relatively new TAA in our mini-array approach, because there are others (for example, p62, p53, MUC, Imp1, Koc, c-myc, survivin, p16, cyclin B1, cyclin D1, and CDK2) that are associated with several types of cancer, such as breast, gastric, lung, and prostate [20,33,34,35].

With the results of our smaller mini-array (panel), the rate of cancer detection is good; therefore, we think that the mini-array has high potential for being taken into consideration for its adoption in a screening program, and that it could be improved by the inclusion of additional tumor antigens. We think that the mini-arrays of specific TAA should be used for different types of cancers, and that customized panels should be rigorously tested for sensitivity and specificity, not only against other tumors, but also against other disease conditions, such as those in autoimmune diseases. The data obtained with this study support previous reports indicating that the detection of serum autoantibodies for the diagnosis of certain types of cancer could be enhanced by using a mini-array comprising several specific TAA or antigens. The autoantibodies against these four TAA could be determined in serum samples qualitatively and/or quantitatively and their levels in blood could be employed as early markers of disease. At present, we are working on the development of a multiplex lateral flow test device by utilizing the four TAA reported herein, in both a qualitative and quantitative manner, and preliminary results to date are promising.

## 5. Conclusions

Our results suggest that TAA mini-arrays may provide a promising and powerful method for improving the detection of breast cancer in women. It must be noted, however, that this is a preliminary study with a small number of patients; thus, it is necessary to conduct a new study with a larger number of patients. TAA in mini-arrays or in lateral flow tests (multiplexing) could be used in screening programs for the diagnosis of breast cancer in high-risk populations.

It is noteworthy that, although the usefulness of the mini-array may be feasible, we do not think that this type of diagnostic test or the use of these TAA and/or of some others will replace the gold standard (test) for breast cancer (mammography), but its possible use as a screening test would be of great impact. On the other hand, we are convinced that the use of some of the TAA reported herein and of others that permit the detection of autoantibodies in the blood of patients, combined with mammography, would significantly increase the sensitivity, specificity early diagnosis of breast cancer.

It can be seen that the sera of four healthy volunteers presented antibodies against some TAAs, two of them very clearly and two lightly, there is the possibility that at least the two with strong signal were in the very early process of tumor formation In such a way that this was not detected in the mammogram, it would be very interesting to give a timely follow-up to these patients.

## 6. Patents

-Patent Number: 324200, México.

-Utility Model: 3842, México.

## Figures and Tables

**Table 1:** Clinical characteristics of subjects with breast cancer and positivity.

Patient	Age (Years)	Stage	Pathology	Tumor Size cm	Ab vs.			
					TPI1	PRDX2	PPIA	A1AT
1	49	T_1_ N_0_ M_0_	ID	<2	+	+	+	+
2	50	T_2_ N_1_ M_0_	IL	>2, <5	+	+	+	+
3	46	T_1_ N_0_ M_0_	ID	<2	−	+	−	+
4	48	T_1_ N_0_ M_0_	ID	<2	+	−	+	−
5	45	T_1_ N_0_ M_0_	ID	<2	+	−	−	+
6	40	T_2_ N_0_ M_0_	ID	>2, <5	−	+	−	+
7	44	T_2_ N_1_ M_0_	ID	>2, <5	−	+	−	+
8	46	T_2_ N_0_ M_0_	IL	>2, <5	+	−	+	−
9	41	T_1_ N_0_ M_0_	IL	<2	−	+	+	+
10	40	T_1_ N_0_ M_0_	IL	<2	+	+	+	+
11	37	T_1_ N_0_ M_0_	ID	<2	+	−	−	+
12	44	T_1_ N_2_ M_0_	ID	<2	+	−	+	+

Ab, antibody; ID, Infiltrating Ductal; IL, Infiltrating Lobular; **+**, positive for protein; **−**, negative for protein.

**Table 2:** Clinical characteristics of subjects without breast cancer and positivity.

Patient	Age (Years)	Stage	Pathology	Tumor Size cm	Ab vs.			
					TPI1	PRDX2	PPIA	A1AT
1	49	T_0_ N_0_ M_0_	N	n/d	**−**	**−**	**−**	**+**
2	43	T_0_ N_0_ M_0_	N	n/d	**−**	**−**	**−**	**−**
3	47	T_0_ N_0_ M_0_	N	n/d	**−**	**−**	**−**	**+**
4	46	T_0_ N_0_ M_0_	N	n/d	**−**	**−**	**−**	**−**
5	50	T_0_ N_0_ M_0_	N	n/d	**−**	**+**	**−**	**−**
6	45	T_0_ N_0_ M_0_	N	n/d	**−**	**−**	**−**	**−**
7	41	T_0_ N_0_ M_0_	N	n/d	**−**	**−**	**−**	**−**
8	47	T_0_ N_0_ M_0_	N	n/d	**−**	**−**	**+**	**−**
9	38	T_0_ N_0_ M_0_	N	n/d	**−**	**−**	**−**	**−**
10	48	T_0_ N_0_ M_0_	N	n/d	**−**	**−**	**−**	**−**
11	46	T_0_ N_0_ M_0_	N	n/d	**−**	**−**	**−**	**−**
12	50	T_0_ N_0_ M_0_	N	n/d	−	−	−	−

Ab, antibody; ID, infiltrating Ductal; IL, Infiltrating Lobular; ***+***, positive for protein, ***−***, negative for protein. n/d, not detected.

**Table 3:** Summary of sensitivity, specificity, and predictive values.

TAA	Sensitivity	Specificity	Positive Predictive Value	Negative Predictive Value	*p* Value
TPI1	1	0.75	0.6667	1	0.0013
					(**)
PRDX2	0.875	0.6875	0.5833	0.9167	0.0272
					(*)
PPIA	0.875	0.6875	0.5833	0.9167	0.0272
					(*)
A1AT	0.8333	0.8333	0.8333	0.8333	0.0033.
					(**)
All	0.8889	0.7333	0.6667	0.9167	<0.0001
					(****)

Table 3. Results by statistical analysis using chi-square and Fisher exact test, with a 95% confidence interval, from the results of immunoreactivity. In addition to p-value, asterisks describe values levels of statistical significance. Since significant (*) to extreme significance (****).
